# Current Landscape of Short‐T2 Imaging Techniques in the Musculoskeletal System: The Past, Present and Future

**DOI:** 10.1002/jmri.29776

**Published:** 2025-04-21

**Authors:** Pranjal Rai, Amit Kumar Janu, Nitin Shetty, Suyash Kulkarni

**Affiliations:** ^1^ Department of Radiology Tata Memorial Hospital, Homi Bhabha National Institute Mumbai India; ^2^ Department of Radiology Advanced Centre for Treatment Research and Education in Cancer (ACTREC) Kharghar Navi Mumbai India

**Keywords:** Black‐bone imaging, CT‐like bone contrast, FRACTURE sequence, short‐T2 imaging, susceptibility weighted imaging, synthetic CT, ultrashort Echo time, zero Echo time

## Abstract

Conventional MRI is limited in imaging tissues with short T2 relaxation times, such as bone, ligaments, and cartilage, due to their rapid signal decay. This limitation has spurred the development of specialized MRI techniques designed specifically for short‐T2 tissue imaging. Traditional pulse sequences, including three‐dimensional gradient echo (3D‐GRE), susceptibility‐weighted imaging (SWI), and Fast Field Echo Resembling a CT using Restricted Echo‐Spacing (FRACTURE), initially addressed some of these challenges but often lacked sufficient resolution or contrast differentiation. Recent advancements, such as ultrashort echo time (UTE), zero echo time (ZTE), 3D‐Bone, and synthetic computed tomography (sCT), have significantly enhanced the diagnostic capabilities of MRI by providing high‐quality, CT‐like visualization without exposure to ionizing radiation. These innovations have substantially improved MRI's ability to depict bone morphology, assess joint pathology, identify subtle fractures, and characterize bone tumors with higher accuracy. Beyond musculoskeletal applications, these techniques have demonstrated emerging clinical utility in additional domains, including pulmonary and dental imaging. This review article evaluates conventional pulse sequences alongside emerging MRI innovations, highlighting their clinical applications, current limitations, and technical considerations. Continued optimization of these techniques promises broader clinical adoption, potentially reducing dependence on invasive and radiation‐intensive imaging modalities.

**Evidence Level:** N/A

**Technical Efficacy:** Stage 3

## 1 Introduction

Imaging of short T2 tissues (bone, tendons, ligaments, menisci, and calcified cartilage) is challenging using conventional MRI techniques due to their extremely short transverse relaxation time (TR) [1]. By the time image sampling begins, the signal from these tissues has already decayed to a minimum, making them appear dark on conventional sequences. This limitation has spurred the development of specialized techniques designed to visualize these short T2 tissues, enabling both their qualitative and quantitative assessments [2, 3].

The pursuit of imaging short T2 tissues is not new and is almost as old as MRI itself. Early imaging techniques in solid‐state NMR were primarily centered on line‐narrowing strategies aimed at reducing spectral line widths. These techniques included Magic Angle Spinning, which rotates the sample at ~54.7° to average out anisotropic interactions, and Radiofrequency (RF) Decoupling, which applies RF pulses to minimize dipolar couplings. By extending T2* relaxation, they enhance spectral resolution and sensitivity, enabling precise molecular quantification and deeper insights into molecular dynamics. Implementing these in vivo was challenging, however, due to rapid sample rotation being impractical for biological systems and high levels of RF power absorption posing safety risks due to excessive specific absorption rate (SAR) and tissue heating. Consequently, short‐T2 imaging in vivo in the current landscape relies heavily on sufficient spatial encoding and data acquisition speed [4].

The ability to image short T2 tissues has important implications for diagnosis, treatment planning, and monitoring of various conditions in clinical practice because it provides valuable information on the morphology and composition of tissues that are generally imaged using other imaging techniques such as radiographs or computed tomography (CT). This may allow reducing the overall radiation dose to the patient while providing CT‐like bone contrast (CLBC) [5, 6]. For example, this may prove useful in the context of osteoarthritis for assessing changes in bone, cartilage, and other joint tissues, which are often difficult to visualize with standard MRI [6]. Dose reduction is also crucial in pediatric imaging, especially for those requiring multiple subsequent imaging sessions. This review article focuses on the history, the current landscape, and ongoing developments in the field of short‐T2 imaging techniques.

## 2 Overview of Short‐T2 Imaging and Generation of CT‐Like Bone Contrast (CLBC)

### 2.1 Conventional MR Imaging Techniques

**FIGURE 1:**
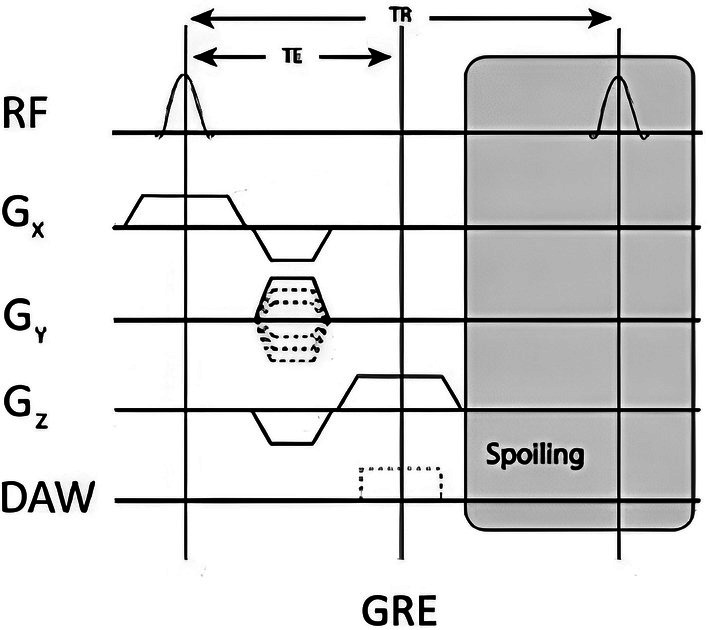
Sequence diagram for Gradient Echo (GRE) sequence. RF: Radiofrequency pulse; Gx: Frequency‐encoding gradient; Gy: Phase‐encoding gradient; Gz: Slice‐selection gradient; DAW: Data Acquisition Window; TE: Echo Time; TR: Repetition Time.

In musculoskeletal MRI, T1‐weighted and proton density (PD)‐weighted sequences are the mainstay for structural bone imaging. Gradient‐echo (GRE) sequences (Figure 1) offer several advantages compared to spin echo (SE) sequences in the context of bone imaging. While being more susceptible to artifacts caused by susceptibility variations, chemical shift, and field inhomogeneity, their primary advantage is the acquisition speed (due to shorter TR), reducing overall scan time, improving patient throughput, and minimizing motion artifacts. Additionally, GRE sequences are more versatile due to their ability to generate different tissue contrasts by manipulating imaging parameters like flip angle (FA), echo time (TE), and TR [7]. This principle has been explored within the context of “black‐bone” imaging, discussed subsequently [8]. Table 1 summarizes vendor‐specific names for 3D‐T1 GRE sequences currently available [9].

**TABLE 1:** Vendor‐specific names used for musculoskeletal 3D MRI imaging.

Sequence	Vendor			
	GE	Siemens	Philips	Toshiba
Bright fluid 3D GRE	SPGR	FLASH	T1‐FFE	T1‐FE
Dark fluid 3D GRE	(VIPR), SSFP, GRASS, DEFT, FIESTA	DESS, TrueFISP, PSIF, GRE	(Balance/T2) FFE	(True) SSFP, FE/PFI
3D FSE	Cube, XETA	SPACE	VISTA	mVox
Dixon technique	IDEAL	Dixon	mDixon	WFOP

**FIGURE 2:**
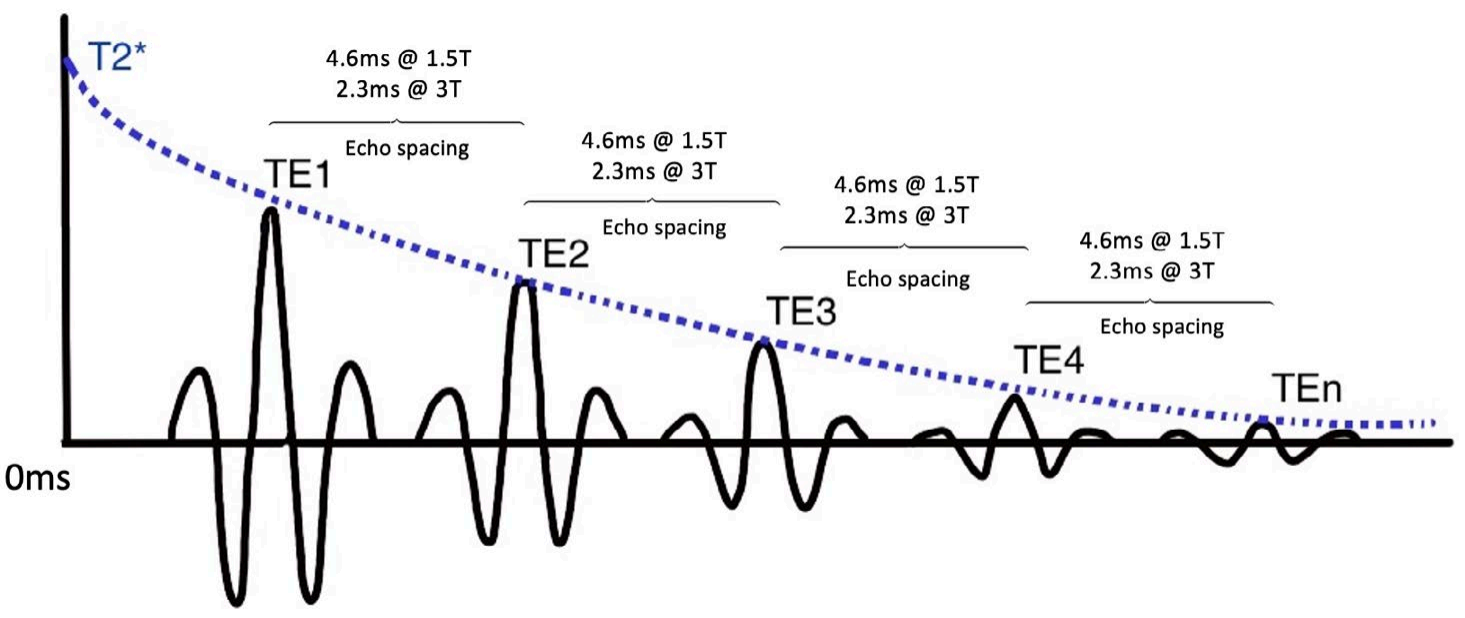
Sequence diagram for Fast field echo Resembling a CT using Restricted Echo‐spacing (FRACTURE). TE1 to TEn represent multiple gradient echoes acquired at specific time points following initial excitation.

Fast Field Echo Resembling a CT using Restricted Echo‐Spacing (FRACTURE) achieves its distinctive CLBC by employing a specifically designed multi‐echo 3D GRE pulse sequence combined with tailored post‐processing methods. Multiple echoes at precise “in‐phase” echo times (e.g., 2.3 ms at 3 T) are acquired, strategically chosen to minimize chemical shift artifacts and reduce T2* dephasing effects at tissue interfaces [10]. (Figure 2) The former improves spatial localization of bone signals, providing sharp delineation of cortical and trabecular bone contours, while the latter mitigates rapid signal decay due to microscopic B_0_ variations at bone‐tissue interfaces, thus preserving signal intensity and enhancing contrast for short‐T2 tissues [11].

Following acquisition, a two‐step post‐processing approach is utilized: first, performing a magnitude summation of all echoes to substantially improve signal‐to‐noise ratio (SNR). Second, by subtracting the last echo image—where short‐T2 tissues exhibit minimal signal—from the summed image, FRACTURE effectively inverts the grayscale intensity relationships. This subtraction step accentuates signal differences, making cortical bone appear dark and soft tissues relatively bright, thus replicating the intuitive bone contrast familiar from CT [12]. Additionally, sequence parameters such as a small FA and short TR are optimized to further enhance tissue differentiation, collectively enabling FRACTURE to provide robust, high‐resolution bone imaging (0.62 mm voxels) suitable for detecting fractures and assessing osseous pathologies [11].

The sequence can be combined with water‐fat separation techniques, such as selecting appropriate in‐phase TEs, to suppress chemical shift artifacts and adipose signal, thereby improving bone delineation and contrast [13, 14]. Additionally, incorporating fat suppression methods, such as water excitation or RF‐based fat saturation pulses, can facilitate more accurate depiction of osseous anatomy while minimizing artifacts [15, 16].

A key benefit is its broad accessibility due to its compatibility with most MRI hardware and software platforms, superior spatial resolution and image contrast compared to alternative CLBC imaging techniques, and diagnostic flexibility due to its 3D isotropic acquisition allowing multiplanar reformatting [17, 18].

However, GRE‐based sequences present certain limitations such as their reliance on T2 weighting for contrast. Susceptibility to variations in magnetic field strength, caused by metal implants, foreign bodies, or iron deposits, can lead to image‐distorting artifacts [10]. The subtraction step used in FRACTURE can also introduce image misregistration, potentially compromising interpretation. Moreover, inversion depicts not only bone as bright but also fluids, ligaments, and tendons, resulting in low specificity and causing difficulty in distinguishing tendon calcifications or avulsion fractures. Finally, these sequences are unable to differentiate between bone marrow edema (BME) and sclerosis, limiting their diagnostic utility in certain cases [19].

A summary of tissues with short‐T2 and their decay times is given in Table 2. The protocol used at our institution for acquiring FRACTURE sequence is summarized in Table 3.

**TABLE 2:** Table summarizing normal adult tissues with the majority of short‐T2 components, and the approximate T2 decay times of each of them.

Amount of short‐T2 components	Tissue or tissue component	Mean T2
Very short (< 1 ms)	Bone	0.4–0.5 ms
	Dentine	0.15 ms
	Enamel	70 μs
Short (> 1 ms)	Periosteum	5–11 ms
	Deep layers of the articular cartilage	5–10 ms
	Knee menisci	5–10 ms
	Ligaments	4–10 ms
	Achilles tendon	0.25 and 0.7 ms, 1.2–0.2 ms, 0.53 ms (88%) and 4.8 ms (12%), 7 ms

**TABLE 3:** Scanning parameters for FRACTURE used at our institute.

Scanner	Ingenia TM (Philips)
Field strength	1.5 T
Sequence	FRACTURE
Repetition time (TR)	11 ms
Echo time (TE)	1.2 ms
Number of excitations (NEX)	2
Flip angle (FA)	8°
Field of view	558 × 558
In‐plane spatial resolution	0.64 × 0.64 mm
Slice thickness	2 mm
Gap	1 mm
Imaging direction	Coronal
SAR	0.037
Fat suppression	No
Scanning time (average)	3 min 12 s

3D‐Bone is the name of another CLBC sequence that has been described [20]. It is also a 3D spoiled GRE without fat suppression, with a short in‐phase TE and modest FA to maximize the contrast of the PD. Using a stack of stars, the k‐space sample is obtained with radial encoding in the in‐plane directions and Cartesian encoding in the through‐plane directions [10].

### 2.2 Applications of 3D‐T1 GRE and FRACTURE

#### 2.2.1 Assessment of Bone Structure

A study comparing FRACTURE and UTE MRI to CT (MVision AI) for visualizing skull bones and fractures found high concordance between FRACTURE and CT across all skull regions. UTE excelled in imaging the skull vault and facial bones, with both MRI sequences showing strong agreement for skull vault fractures [21].

A pilot study by Ryu et al. found that CT and FRACTURE sequences provided the clearest views of the intertubercular groove and outperformed T1W, T2W, and PDW images in visualizing the trabecular pattern and tubercles in the canine shoulder [22].

3D T1 VIBE sequences have also been proven to be comparable to CT in detecting intra‐cortical fracture lines in Medial Tibial Stress Syndrome [23].

Dixon 3D‐dual‐TE T1W‐FLASH (with water‐only data) has shown comparable results to 3D CT osseous models of the shoulder in assessing glenoid and humeral measurements, including glenoid width and height, humeral head dimensions, and biceps groove width [24]. Moreover, Dixon 3D‐T1W‐FLASH‐based MR shoulder reconstructions have been validated against arthroscopy for assessing glenoid bone loss, revealing no significant differences [25].

Lansdown automated the manual segmentation of CLBC 3D FLASH using 3D Slicer, showing strong inter‐modality agreement (ICC = 0.94–0.99) and less than 2% variation in 88% of measurements between MRI and CT, with bone loss estimates differing by 0%–6% [26].

#### 2.2.2 Assessment of Articular Cartilage

Modern GRE sequences provide high‐resolution, isotropic or near‐isotropic imaging for articular cartilage assessment. Contrast weighting of HiRes‐3D GRE can be classified into dark fluid and bright fluid signal sequences [27]. HiRes‐3D GRE sequences have shown lower sensitivity and greater specificity compared to 2D FSE sequences for the evaluation of cartilage defects. This is partially due to the increased susceptibility associated with the 3D GRE sequences, which reduces the conspicuity of subchondral marrow signal changes [28, 29].

Various studies comparing HiRes‐3D FSE and GRE sequences for articular cartilage evaluation have shown that sequences with higher fluid‐to‐cartilage contrast ratios yield superior diagnostic performance, despite having inconsistent results regarding diagnostic efficacy and reader‐perceived visual preference [30, 31].

#### 2.2.3 Assessment of Joint Pathologies

FRACTURE has closely matched CT for assessing glenoid bone loss, aiding preoperative planning of joint fractures, identifying congenital abnormalities like calcaneonavicular coalition, and detecting subtle bone lesions [11].

Researchers evaluated 3D‐T1 GRE, UTE, and FRACTURE MRI for detecting osseous pathologies in patients with suspected shoulder dislocations, comparing these to traditional CT imaging. All three MRI sequences reliably identified osseous abnormalities, with UTE showing the best correlation to CT for measurements on CT‐like images [32].

3D T1 VIBE MR arthrography has also proven to be a viable alternative to traditional T1‐Fat saturated sequences for detecting significant articular‐sided tears [33]. Tian et al. found fat‐suppressed 3D VIBE sequences highly effective for identifying traumatic glenoid lesions, with 95.0% sensitivity and 93.8% specificity for glenoid bone loss, and 95.7%–100% sensitivity and 93.9%–97.0% specificity for bony Bankart lesions compared to multislice CT [34].

Stillwater et al. compared osseous reformats of 3D VIBE and CT in patients with glenohumeral instability and found that both performed similarly, with no significant differences in glenoid height, humeral height, or glenoid width measurements [35].

The Dixon 3D FLASH sequence has shown 100% agreement with CT for diagnosing and locating cam deformities in femoro‐acetabular impingement. It achieved 89.5% agreement for anterior‐inferior iliac spine characterization and 64.7% for neck‐shaft angle and LCEA measurements. Over 4 years, 3D‐MRI saved an average radiation dose of 3.09 mSv per patient, totaling a reduction of 479 mSv [14].

FRACTURE has also demonstrated high consistency with radiographs and CT for evaluating knee and ankle joint pathologies [36].

#### 2.2.4 Spine Imaging

**FIGURE 3:**
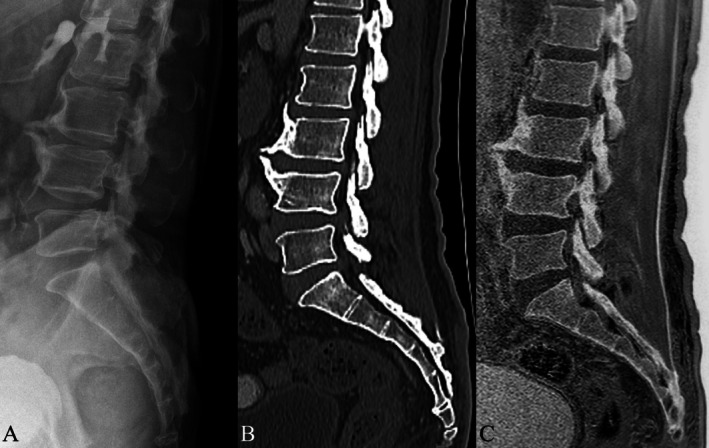
Lateral lumbar radiograph (A) with sagittal reformatted CT (bone window) (B) and FRACTURE MRI (C) demonstrating anterior L3 and L4 osteophytes.

3D T1W spoiled GRE has demonstrated higher diagnostic performance and image quality than UTE MRI for detecting vertebral fractures and degenerative bone changes (Figure 3) (such as osteophytes, sclerosis, and spondylolisthesis), with stronger agreement to CT [17]. Adding FRACTURE or UTE to standard MR sequences has shown to deliver comparable information regarding osseous cervical spine status when compared to CT [8]. 3D T1 VIBE sequences have matched CT in sensitivity and specificity for detecting complete pars fractures (up to 100% accuracy), while also being highly effective for incomplete fractures [16].

FRACTURE has been evaluated against CT and MRI for assessing craniocervical junction in dogs, showing that CT and single‐echo FRACTURE (sFRACTURE) provided superior cortical and trabecular bone visibility and higher cortical bone‐muscle contrast compared to MRI, whereas multi‐echo FRACTURE (mFRACTURE) demonstrated lower contrast between cortical and trabecular bone [12].

#### 2.2.5 Assessment of Bone Tumors

**FIGURE 4:**
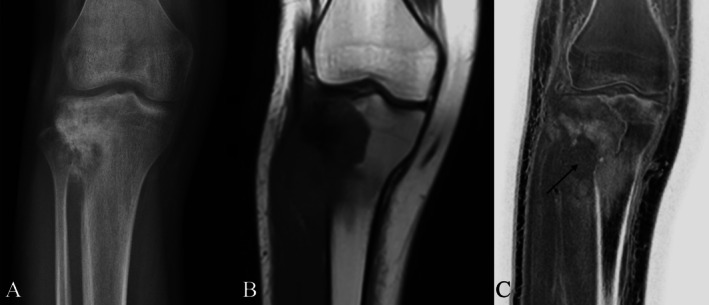
Radiograph of the knee (A), Coronal T1 MRI (B) and FRACTURE MRI of the knee (C) in a patient with proximal tibial osteogenic sarcoma. Note the extraosseous soft tissue better appreciated on T1 and FRACTURE MRI (arrow). Surrounding bone marrow edema is also seen on T1 as hypointensity, which is represented by sclerosis on FRACTURE MRI.

**FIGURE 5:**
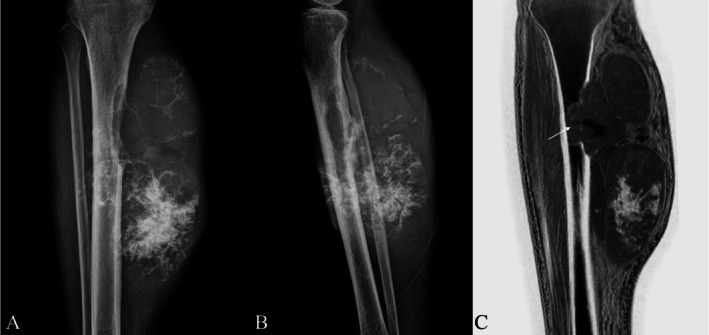
Anteroposterior (A) and Lateral (B) radiographs of the right tibia showing a large expansile mass with rings‐and‐arc type calcification with extraosseous soft‐tissue component arising from the diaphysis. Corresponding FRACTURE sequence (C) clearly demonstrates the intramedullary extent of the mass (white arrow).

**FIGURE 6:**
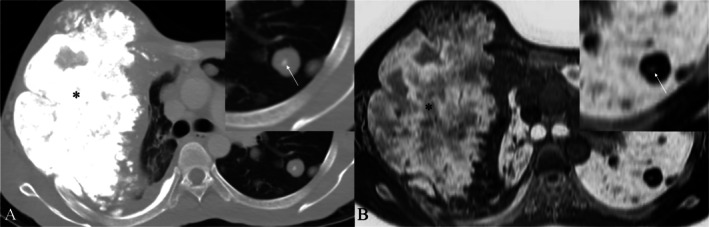
Axial non‐contrast CT, lung window (A) shows a large mass arising from the right chest wall (asterisk) with corresponding FRACTURE sequence (B) demonstrating the osseous matrix within the mass. Also note the tiny calcification seen within the metastatic lung nodules (arrows), characteristic of an osteosarcoma, seen clearly on both images.

Gersing et al. in their pilot study evaluated the diagnostic utility of CT‐like images derived from MRI and simulated radiographs in comparison to conventional radiographs (CR) for assessing benign and malignant bone tumors. Additional information on soft‐tissue extension was better appreciated on MR‐derived CT‐like images (Figures 4, 5, 6) compared with CR; the sensitivity of both modalities for the final diagnosis of the lesion was high; and there was a substantial agreement between CR and CT‐like images along with simulated radiographs [18, 37].

### 2.3 Susceptibility‐Weighted Imaging

Widely employed in the imaging of brain microbleeds and microvasculature, susceptibility‐weighted imaging (SWI) combines a velocity‐corrected 3D GRE pulse sequence with particular post‐processing techniques, thus enhancing T2* susceptibility contrast [38, 39].

**FIGURE 7:**
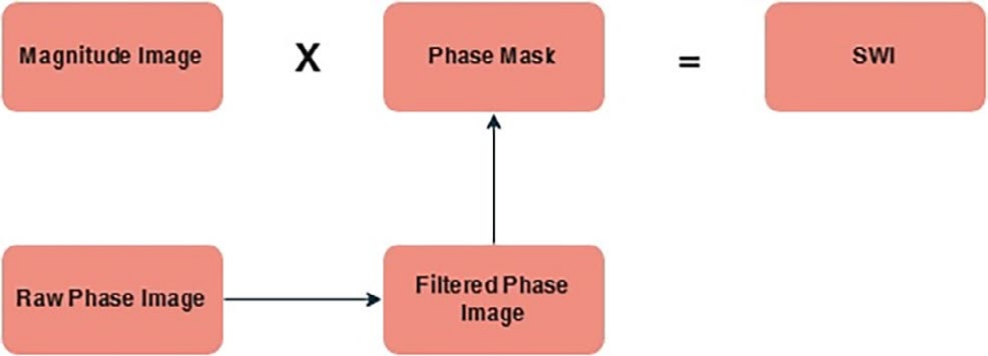
Schematic representation of synthetic CT generation using susceptibility‐weighted imaging (SWI).

Four sets of images are displayed after post‐processing: magnitude, high‐pass filtered phase, combined magnitude/phase, and thick minimum intensity projections. Calcification and bone are diamagnetic, which distorts the local magnetic field. They can be distinguished from paramagnetic compounds, like hemosiderin, which alter phase in a different way on filtered phase pictures. With thicker through‐plane voxels than other CLBC approaches, these use a low‐angle T2*‐weighted 3D GRE pulse sequence with lower TEs (14–20 ms) than usually used for brain SWI. CLBC MR images are obtained by applying gray‐scale image inversion. While SWI images are not employed for the primary assessment of osseous structures, they can occasionally be useful in differentiating between paramagnetic and diamagnetic materials [20]. Figure 7 shows a schematic representation of synthetic CT generation using SWI.

### 2.4 Applications of SWI

#### 2.4.1 Assessment of Bone Structure

SWI has demonstrated superior reliability compared to anteroposterior radiographs for hip morphological measurements, including Sharp's angle (*R* ^2^ = 0.80 vs. 0.37), Tönnis angle (*R* ^2^ = 0.86 vs. not measurable), Wiberg's lateral center‐edge angle (*R* ^2^ = 0.88 vs. 0.40), and caput‐collum‐diaphyseal angle (*R* ^2^ = 0.38 vs. 0.18) [40].

#### 2.4.2 Assessment of Joint Pathologies

Ulas et al. compared SWI, T1W, and VIBE MRI for detecting peripheral arthritis, using CT as the reference. All MRI sequences had high sensitivity (94%–100%), with SWI having the highest specificity (90%). SWI's sum score was similar to CT, while T1W and VIBE produced significantly higher scores [41].

SWI has outperformed standard MRI and radiographs for evaluating sub‐coracoacromial spurs in patients with suspected subacromial impingement syndrome (SAIS), offering high sensitivity, specificity, and accurate spur size measurement [42].

Using CT as the reference standard, SWI has demonstrated superior diagnostic performance compared to standard T1 SE sequences for detecting sacroiliac joint (SIJ) structural abnormalities in patients with axial spondyloarthritis [43].

#### 2.4.3 Spine Imaging

Böker compared SWI to conventional MRI for vertebral body fractures using CT as the reference, finding SWI superior in detecting cortical breaks, fracture lines, and posterior bone involvement, with a sensitivity of 0.86–0.98 and a specificity of 0.99–1.00 [44].

SWI has demonstrated technical superiority in evaluating degenerative spinal conditions, particularly in differentiating sclerotic from non‐sclerotic Modic changes, achieving a sensitivity of 100% and specificity of 95% [45]. Additionally, SWI effectively delineates sclerotic vs. non‐sclerotic Modic changes (100% sensitivity; 95% specificity) while accurately quantifying degenerative lesions like osteophytes and disc herniations, providing results comparable to CT and radiographs [46, 47].

#### 2.4.4 Assessment of Bone Tumors

**FIGURE 8:**
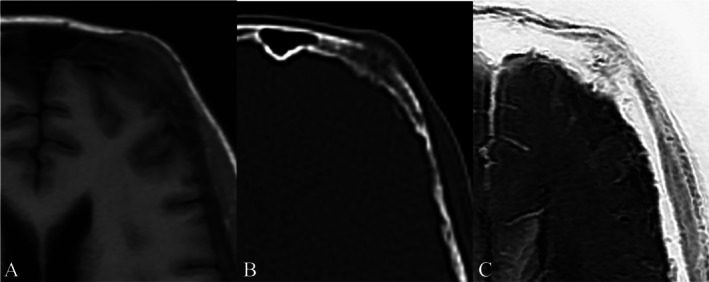
Axial non‐contrast T1‐weighted MRI (A) demonstrates a metastatic soft‐tissue lesion involving the left frontal bone in a patient with lung adenocarcinoma. Corresponding axial CT in bone window (B) and grayscale‐inverted susceptibility‐weighted imaging (SWI) (C) confirm associated osteolytic changes.

SWI has demonstrated superior performance compared to conventional MRI in evaluating spinal metastases, exhibiting a strong correlation with CT in assessing both lesion size and the degree of mineralization [48]. (Figure 8).

### 2.5 Ultrashort Echo Time (UTE) and Zero Echo Time (ZTE)

Modern MRI scanners have facilitated the imaging of short‐T2 tissues through the development of multiple short‐TE acquisition methods. These advancements leverage the capabilities of modern scanners, including:

1. Rapid RF switching: Achieved in tens of microseconds, allowing for efficient manipulation of RF pulses.
2. Suitably short RF pulses: Minimizing signal losses due to T2 relaxation during pulse application.
3. Minimum delay digital filtering: Enabling rapid signal processing and reducing data acquisition times.
4. Fast sequence repetition: Allowing for repeated signal acquisition within a shorter timeframe [49].

**FIGURE 9:**
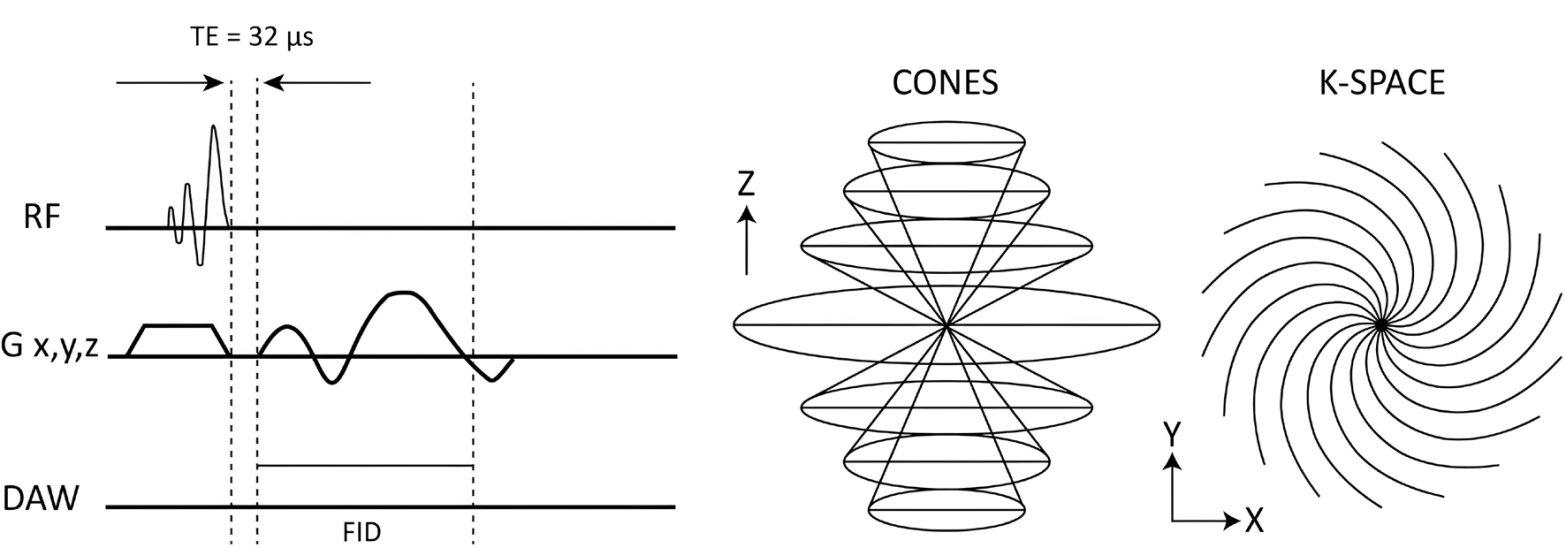
Sequence diagram for 3D Ultrashort Echo Time (UTE) where a short slab‐selective RF pulse is used for signal excitation, following which a Cones trajectory k‐space sampling is applied.

Two‐dimensional (2D) UTE sequences achieve very short TE (around 8 μs) by using half‐pulse excitation and a radial sampling [50]. Likewise, UTE imaging with 3D capabilities can be obtained by combining short, hard excitation pulses with 3D spiral or radial sampling techniques (Figure 9) [51, 52]. Instead of these short, hard pulses, longer, frequency‐modulated pulses can also be used, and this concept is referred to as Capturing Signals from Fast‐relaxing Spins with Frequency Swept Imaging With Fourier Transformation (SWIFT). Supplemented by efficient long‐T2 suppression techniques, we can obtain comparable SNR and contrast‐to‐noise (CNR) ratios to the conventional sequences [53].

**FIGURE 10:**
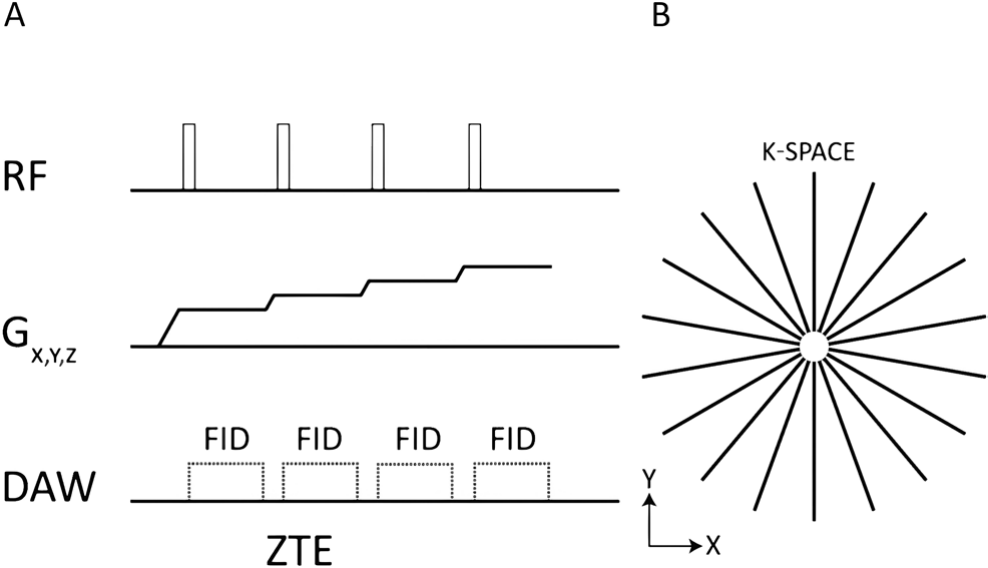
Sequence diagram for 3D Zero Echo Time (ZTE) where a non‐selective rectangular RF pulse is applied for a short duration (A) followed by a 3D center‐out radial k‐space sampling technique with some missing points in the center (white area in B). Readout gradients are turned on before signal excitation and evolve incrementally (Gx, y and z in A). DAW: Data Acquisition Window; RF: Radiofrequency; FID: Free Induction Decay.

Further propagating this same principle, ZTE can be achieved by switching on the projection gradient *before* the RF pulse, enabling encoding of the newly generated transverse magnetization at full strength and without delays. TE is thus essentially nil, and complete k‐space speed is available immediately. This wide‐band hard‐pulse zero‐TE approach is referred to as ZTE imaging [3]. (Figure 10).

ZTE sequences inherently acquire 3D data due to the presence of a projection gradient during excitation, which does not allow for selective imaging of a single slice. To ensure consistent signal across the entire field of view (FOV), the high amplitude RF pulse needs to uniformly excite all frequencies within the gradient's range, avoiding FA variations across the same location depending on the gradient direction [3]. To create images with almost negligible TE, ZTE uses algebraic reconstruction, extrapolation of oversampled recordings, and radial k‐space filling [3, 49, 54].

Additional capabilities include its virtually silent acoustic operation stemming from the fact that the projection gradient does not need to be switched off, but only gradually re‐oriented [55]. Decrease in the eddy effects due to minimal gradient changes also makes the imaging highly robust and very time‐efficient [49].

The high‐bandwidth excitation pulses required for uniform coverage can lead to increased energy deposition, consequently raising the SAR, particularly pronounced at higher B_0_ [49]. Additionally, the rapid TE decay results in reduced contrast, as the signal primarily reflects spin density rather than T2 relaxation differences. Furthermore, it is susceptible to artifacts arising from extremely short‐lived MR signals generated by metallic implants or RF hardware. Finally, the lack of a dedicated readout dimension necessitates an increase in the number of projections to enlarge the FOV in any direction, which may extend scan time [49].

### 2.6 Applications of UTE and ZTE

#### 2.6.1 Assessment of Bone Microstructure

Cortical bone water (BW) constitutes about 20% water by volume and can serve as a more sensitive predictor of osteoporosis than volumetric bone density. UTE/ZTE imaging has been effectively utilized for the in vivo quantification of cortical BW, providing a sensitive and non‐invasive approach to assessing bone quality beyond conventional density measurements [56].

ZTE has enabled direct in vitro visualization of trabecular bone microstructure by imaging matrix‐bound water, providing MR images with structural accuracy superior to μCT and CR [57]. Other similar preclinical and translational studies have evaluated the use of UTE imaging as a sensitive biomarker of subtle microstructural and functional bone properties, revealing strong correlations between UTE‐derived parameters (e.g., macromolecular PD, collagen proton fraction, and water PD) and μCT measured bone porosity and mineral density [58, 59].

#### 2.6.2 Assessment of Joint Pathologies

ZTE MRI has significant utility in peripheral as well as central joint imaging. In the shoulder, it accurately grades glenoid morphology, detects Bankart and Hill‐Sachs lesions, subchondral cysts, osteophytes, and fractures, often eliminating the need for concurrent CT [60]. In knee imaging, ZTE enhances the detection of early osteophytes in osteoarthritis and delineates osteochondral junction abnormalities, especially when combined with inversion recovery fat saturation or deep learning reconstruction [61]. For the hip, it facilitates accurate assessment of femoroacetabular impingement, dysplasia, and traumatic injuries with results comparable to CT [62]. ZTE MRI is similarly beneficial in ankle and foot trauma by clearly defining fractures and anatomical variants [63]. In the wrist and hand, it effectively visualizes mature calcifications, ossifications, and subtle lesions like calcific tendinitis and enchondromas [37, 64]. Lastly, in the temporomandibular joint, ZTE offers comparable diagnostic accuracy to cone‐beam CT for identifying osseous changes [65].

ZTE is also highly valuable for assessing sacroiliitis, a hallmark of spondyloarthropathies, demonstrating comparable or superior sensitivity to low‐dose CT in detecting structural lesions such as erosions, sclerosis, and joint space changes in the SIJ, making it a radiation‐free alternative for evaluating conditions like ankylosing spondylitis (AS) and enthesitis‐related arthritis [66, 67, 68, 69, 70].

#### 2.6.3 Spine Imaging

In the cervical spine, ZTE enhances the depiction of cortical bone, facilitates accurate measurement of neural foraminal stenosis and foraminal diameter, and detects ossification of the posterior longitudinal ligament (OPLL). For the lumbar spine, ZTE effectively identifies degenerative changes, compressive fractures, osteophytes, and cortical abnormalities, serving as a viable alternative to CT and complementing conventional MRI for comprehensive evaluation [21, 71, 72, 73, 74].

#### 2.6.4 Assessment of Bone Tumors

**FIGURE 11:**
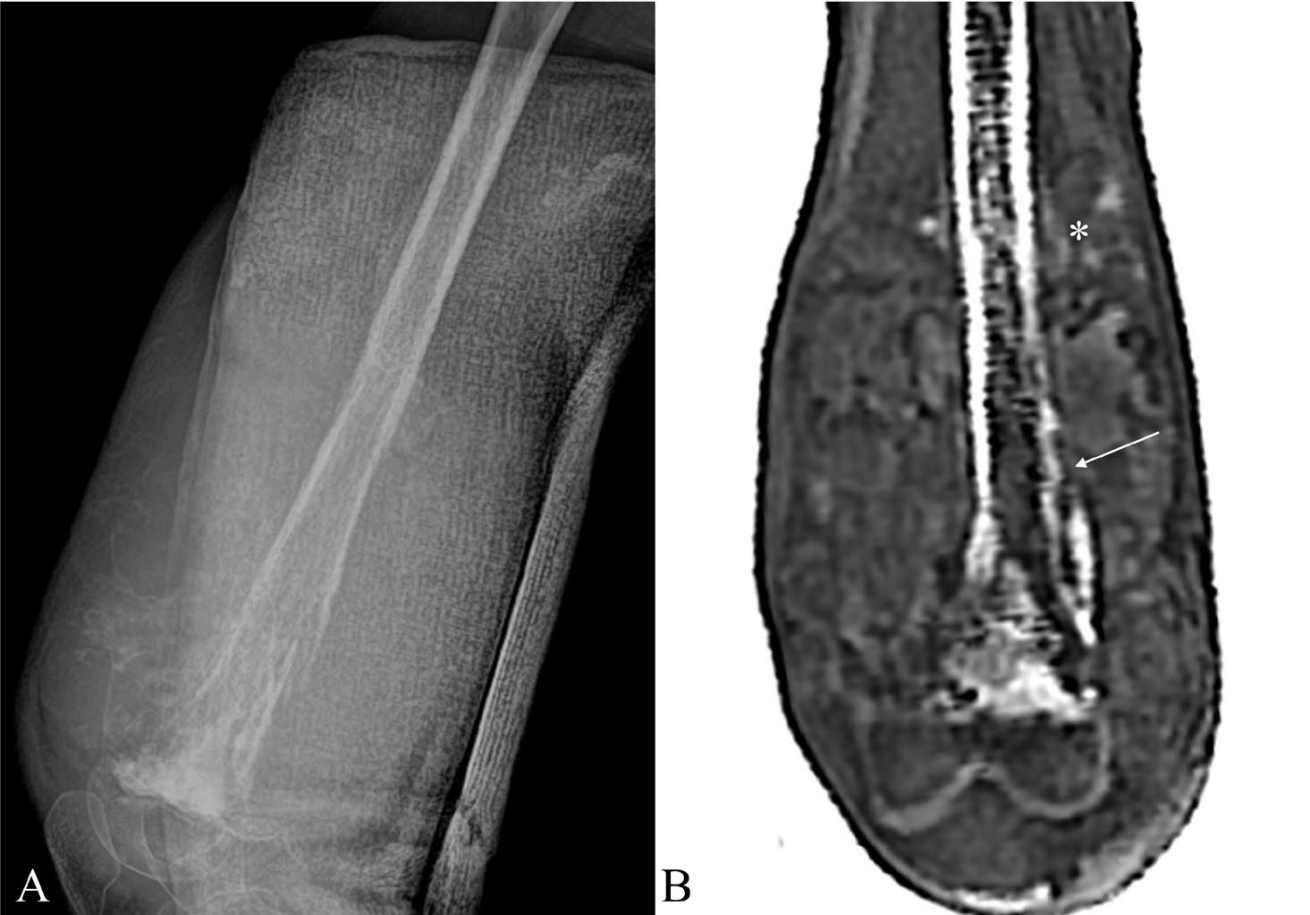
Anteroposterior radiograph (A) demonstrates an aggressive lesion involving the distal femoral diaphysis with associated periosteal reaction and soft‐tissue involvement. Corresponding zero echo time (ZTE) image (B) clearly depicts cortical breach (arrow) and adjacent soft‐tissue matrix mineralization (asterisk), further delineating the extent of disease.

Recent studies have also highlighted the potential of ZTE MRI for oncologic imaging. Lecouvet et al. demonstrated ZTE's capability in whole‐body detection of osteolytic lesions, closely matching CT in diagnostic accuracy, emphasizing its clinical value for identifying metastatic disease and other osteolytic processes [75]. Additional research confirms ZTE's effectiveness in clearly delineating tumor boundaries, periosteal reactions, sclerosis, calcifications, and areas of bone destruction, comparable to CT [76, 77]. (Figure 11).

#### 2.6.5 Hybrid imaging and attenuation correction

Hybrid Positron Emission Tomography (PET)/MR imaging incorporates the use of MR‐based attenuation correction to determine and compensate for the photon energy attenuation introduced by the soft tissues within the patient. This attenuation correction is accomplished by PET/CT scanners by adapting the X‐ray attenuation information provided by the CT. In contrast to this, this attenuation correction in PET/MR imaging is accomplished by the segmentation of the MR data to identify different tissue classes. The accuracy of the reconstructed PET data will thus be determined by the accuracy of this segmentation.

ZTE enables PET attenuation correction by accurately depicting bone, facilitating two primary approaches: segmentation‐based methods and pseudo‐CT (pCT) generation. Segmentation‐based correction uses ZTE images to segment tissues (air, soft tissue, bone) and assigns predefined attenuation coefficients at 511 keV, resulting in anatomically accurate attenuation maps superior to traditional dual‐echo UTE methods. Alternatively, ZTE images can be transformed into CT‐like pseudo‐CT images through intensity normalization, bias correction, and advanced deep learning (DL) algorithms, generating synthetic Hounsfield unit (HU) maps suitable for attenuation correction (similar to synthetic CT, discussed later). This has been explored across multiple anatomies, including the head and the pelvis [78, 79]. Future research applications may extend its application to whole‐body PET/MR, combining segmentation methods, truncation completion, and atlas‐based approaches to enhance overall imaging quality [80].

### 2.7 Synthetic CT (sCT)

**FIGURE 12:**
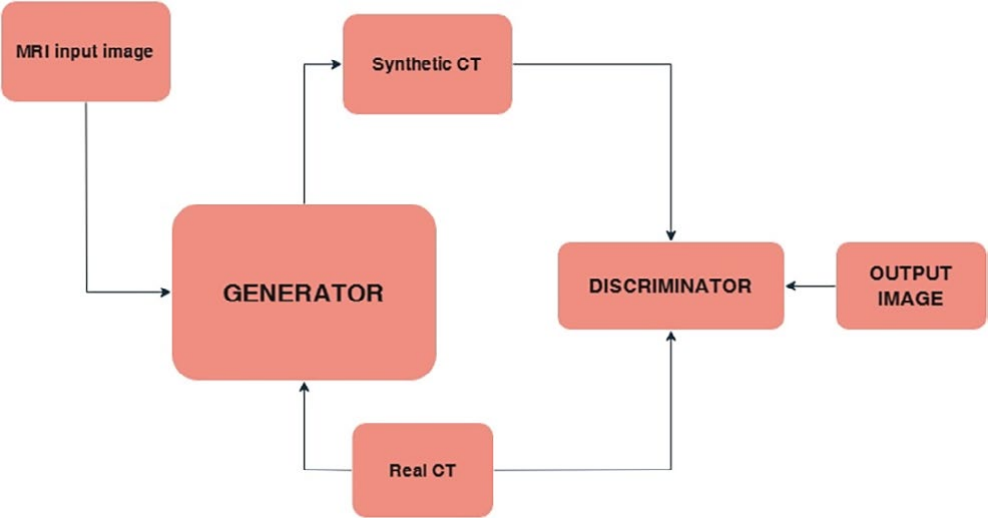
Schematic representation of synthetic CT generation using Generative Adversarial Networks (GANs).

DL has revolutionized the field of generating CLBC from MRI due to its ability to learn complex non‐linear relationships between MRI signals and CT attenuation values. It involves training artificial neural networks, such as Convolutional Neural Networks (CNNs), often utilizing architectures like U‐Net or Generative Adversarial Networks (GANs), on large datasets of paired MRI and CT scans (Figure 12) [81]. These networks learn to map the intricate features present in MRI data to the corresponding HU values in CT images. Once trained, the model can generate sCT images from new MRI data of similar anatomy. Various studies have explored the use of different input MRI sequences, including T1‐weighted, T2‐weighted, Dixon images, UTE, and ZTE sequences, either individually or in combination, to optimize sCT quality for specific anatomical regions and clinical applications [20, 78].

Several commercially available sCT technologies exist, such as BoneMRI (MRIguidance), MVision AI, and RT Image Suite (Siemens Healthcare). These often utilize proprietary DL algorithms trained on extensive datasets. Some of these may involve cloud‐based post‐processing [81].

Despite advancements, sCT faces several limitations. Diagnostic accuracy remains inferior to conventional CT given the paucity of training data for subtle bone abnormalities and soft‐tissue calcifications, necessitating review of source MRI data such as ZTE images. Algorithm performance varies by anatomical region and specific DL model [81, 82]. Motion and susceptibility artifacts (e.g., from dental amalgam) can compromise MRI inputs and sCT quality. Implementation requires specialized infrastructure, stable MRI scanner performance, and DL expertise [83]. Lack of standardized, vendor‐neutral guidelines leads to variability in clinical practice. DL methods may introduce unrealistic “hallucinations,” highlighting the need for automated detection methods [84].

### 2.8 Applications of sCT

#### 2.8.1 MR‐Only Radiotherapy Planning

CT traditionally provides the electron density information [in HU], necessary for accurate radiation dose calculation in radiotherapy treatment planning. sCT aims to replace the planning CT by providing estimated HU values that can be used for dose computation. Studies have validated the dosimetric accuracy of sCT in various anatomical regions, including the brain, head and neck, pelvis, and abdomen, and even for applications like Gamma Knife radiosurgery [78, 85, 86, 87, 88]. This can eliminate the need for a separate CT simulation scan, thereby reducing the overall radiation exposure to patients and streamlining the treatment planning process.

#### 2.8.2 Evaluation of Bone Morphology and Pathology

sCT can provide high‐quality volumetric rendering of osseous structures. Studies have shown the equivalency of MRI‐based sCT to CT for geometric measurements in the lumbar spine and hip [89, 90]. It has shown potential for detecting sclerosis and ankylosis of the SIJ in AS patients [91]. In children with rare musculoskeletal diseases, sCT has accurately identified intraosseous lesions and heterotopic ossification, with HU measurements correlating with bone density [92].

Table 4 summarizes the commonly used techniques for CT‐like bone imaging, their advantages, and limitations (Chong et al.) [20].

**TABLE 4:** A summary of the commonly used techniques for CT‐Like Bone Contrast imaging using MRI.

	Joint imaging	Bone morphology imaging	Spine imaging	Bone tumor imaging	Pulmonary imaging	Dental imaging	Attenuation correction
3D‐T1 GRE	Cartilage loss in joints, Craniocervical junction and shoulder imaging in canines, knee, ankle and shoulder imaging in humans, traumatic joint dislocation, glenoid bone loss, FAI	Fractures, intra‐cortical fracture lines in MTSS	Vertebral fractures, degenerative changes	Differentiation of benign and malignant bone tumor morphologies, soft‐tissue component	—	—	—
SWI	Subacromial spurs, Arthritis, Sacroiliac joint imaging	Hip morphology	Vertebral fractures, Neuroforaminal stenosis and other degenerative changes	Detection of osteolytic and osteoblastic spinal metastases	—	—	—
UTE and ZTE	Shoulder imaging, Sacroiliac joint imaging	Cortical bone water estimation, Trabecular bone microstructure	Neuroforaminal stenosis	—	Lung morphology, Restrictive and obstructive lung diseases, Nodules, consolidations, bronchopulmonary dysplasia	Evaluation of Enamel and Dentine layers, Early demineralization and caries lesion	Hybrid imaging
s‐CT	Sacroiliac joint imaging	Hip and lumbar spine morphology	—	Planning treatment using MRI‐guided focused ultrasound, Detection of intraosseous lesions in children	—	—	Radiation therapy and Gamma Knife Radiosurgery planning

Table 5 summarizes the various use cases for different CLBC imaging techniques.

**TABLE 5:** Overview of the different modalities used for “CT‐like” MR imaging and their utility in the assessment of various aspects of musculoskeletal imaging.

	T1wGRE	SWI	UTE	ZTE	Deep learning	3D‐Bone
Technique	Imaging cortical bone indirectly. RF flip angle optimization and greyscale inversion with a short TE/TR	Imaging cortical bone indirectly. Low flip angle and intermediate TE T2*W 3D GRE and greyscale inversion used to generate magnitude images.	Imaging cortical bone directly. Short RF pulses with data acquisition while readout gradient is turned on.	Imaging cortical bone directly. Readout gradient applied before RF pulse, thus avoiding the need for switching between TRs.	Extension of 3D T1w‐GRE. Multi‐echo Dixon GRE and CT images used for training the deep learning model, to generate CLBC synthetic CT images.	Imaging cortical bone indirectly. Small flip angles and greyscale inversion in a low‐TE in‐phase proton density stack‐of‐stars 3D GRE pulse sequence
Advantages	Commonly available, Low MRI hardware/software requirements, post‐processing to yield 3D CLBC images. Good depiction of soft tissues.	Commonly available, Low MRI hardware/software requirements, Post‐processing automatically to produce magnitude images	Post‐processing to generate 2D and 3D CLBC images. Provide some quantitative tissue parameters such as T2*. More flexible contrasts than ZTE. More flexible contrasts than ZTE.	Post‐processing to generate 3D CLBC images. Provide some quantitative tissue parameters such as T2*. More flexible contrasts than ZTE. Shorter scan times and higher SNR than UTE Less sensitive to gradient imperfections than UTE. Low acoustic noise for patient comfort	Low MRI hardware/software requirement. Off‐line image reconstruction software/hardware. Provides Hounsfield unit maps for cortical bone due to its high specificity.	Commonly available. Low MRI hardware/software requirements. No post‐processing needed. Robust to motion artifacts.
Disadvantages	No quantitative tissue parameters. Severe metal susceptibility artifacts. Moderately prone to motion artifacts.	No quantitative tissue parameters. Severe metal susceptibility artifacts. Moderately prone to motion artifacts.	Not generally available, High MRI hardware/software requirements. Moderate metal susceptibility artifacts. Prone to motion artifacts. Sensitive to off‐resonance blurring.	Not generally available, High MRI hardware/software requirements. Moderate metal susceptibility artifacts, Prone to motion artifacts.	Limited availability. Prone to severe metallic susceptibility artifacts and moderate motion artifacts.	No quantitative tissue parameters. Prone to severe metallic susceptibility artifacts.
Vendor‐specific names	VIBE, 3D FLASH, FRACTURE, 3D SPGR, 3D T1‐FFE, THRIVE<, FFE 3D Single‐Echo and Multi‐Echo, FE 3D Single‐Echo and Multi‐Echo	SWI	ZTE, UTE, PETRA, FFE3D with UTE Single‐Echo and Multi‐Echo		BoneMRI	StarVIBE, 3D VANE XD, LAVA Star

## 3 Advancements and Future Prospects

Ongoing advancements in short T2 MRI aim to overcome existing limitations and significantly expand clinical applications. Future improvements in UTE will focus on novel k‐space trajectories and sampling schemes for better SNR and reduced artifacts, integration of contrast preparations [e.g., Inversion Recovery (IR)‐UTE, UTE‐off‐resonance saturation (OSC), UTE‐magnetization transfer (MT)], and sophisticated multi‐echo approaches for quantitative T2* mapping of tissues like cartilage [52, 53]. ZTE imaging will likely address current challenges such as spatial resolution and center k‐space sampling efficiency through innovations in pulse encoding, sweep pulse excitation, and hybrid approaches like ZTE—pointwise‐encoding and time reduction with radial acquisition (PETRA) [93]. Moreover, emerging hybrid techniques combining ZTE's rapid acquisition with UTE's contrast flexibility, along with developments in sequences like SWIFT and Water‐ and Fat‐Suppressed Proton Projection MRI (WASPI), promise enhanced image quality, efficiency, and novel diagnostic capabilities [2].

Future scanners with improved RF transmission and reception capabilities, faster and more efficient T/R switching, and enhanced console specifications are vital for pushing the boundaries of short T2 imaging, particularly for achieving even shorter TEs and higher bandwidths necessary to visualize extremely short T2 components. Higher field strength (e.g., 7 T) may also play a role in improving SNR and potentially enhancing contrast in short T2 tissues [94].

Future research may focus on developing contrast agents specifically tailored to enhance visualization of short T2 tissues using UTE/ZTE imaging. One promising direction involves iron oxide nanoparticles (IONP), which can generate positive contrast through T1 shortening rather than conventional T2* effects, potentially improving the imaging of short T2 tissues [4]. DL reconstruction algorithms are being applied to ZTE data to improve image quality by identifying and reducing noise and artifacts. These techniques accelerate image acquisition by reconstructing images from undersampled k‐space data, which is particularly beneficial for ZTE, which can have longer acquisition times, especially when aiming for high resolution [95, 96].

**FIGURE 13:**
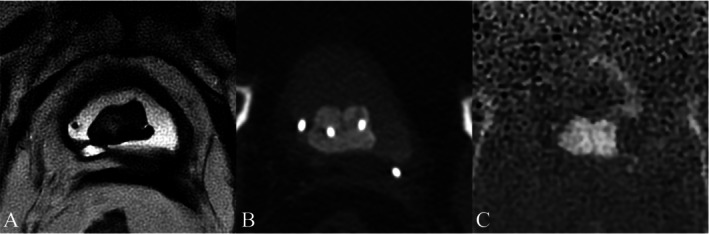
Axial T2‐weighted MRI (A) demonstrates a hypointense lesion within the urinary bladder at the distal tip of a DJ stent in a patient previously treated for cervical squamous cell carcinoma. Corresponding axial CT in bone window (B) and zero echo time (ZTE) MRI (C) confirm dense calcification within the lesion, consistent with a bladder calculus.

Although musculoskeletal imaging remains the primary focus, short T2 MRI techniques have expanded applications across multiple domains. Pulmonary imaging using UTE and ZTE holds promise for improved lung assessment without radiation, including detailed evaluation of parenchymal structures [97, 98, 99]. ZTE's high‐resolution depiction of dental tissues enables early diagnosis of dental pathologies, like caries, without ionizing radiation, stimulating further hardware and sequence optimizations [54]. ZTE has also shown promise for detecting urinary stones without radiation exposure (Figure 13) [100]. Despite technical challenges, short T2 methods are increasingly applied in neuroimaging to better visualize tissues previously difficult to image and in vascular imaging to capture turbulent flow and small vessels more clearly [50]. Beyond medical applications, short T2 MRI offers substantial potential in materials science, non‐destructive testing, and the food industry, leveraging its unique capability to image materials with rapid signal decay [4].

In conclusion, advancements in short‐T2 MRI techniques, including UTE, ZTE, and DL‐driven synthetic CT, have allowed modern MR scanners to provide CLBC, facilitating precise diagnosis and monitoring of various musculoskeletal conditions, while simultaneously reducing patient exposure to ionizing radiation. Broader applications like pulmonary and dental imaging highlight their expanding utility. Nevertheless, technical challenges persist, such as spatial resolution limitations, vulnerability to motion and susceptibility artifacts, and the requirement for specialized equipment and DL expertise. Future research directions to enable broader clinical adoption involve optimizing imaging sequences for improved resolution and reduced artifacts, developing targeted contrast agents, and establishing standardized clinical protocols.

## References

[1] K. Y. Cheng , D. Moazamian , Y. Ma , et al., “Clinical Application of Ultrashort Echo Time (UTE) and Zero Echo Time (ZTE) Magnetic Resonance (MR) Imaging in the Evaluation of Osteoarthritis,” Skeletal Radiology 52, no. 11 (2023): 2149–2157, 10.1007/s00256-022-04269-1. 36607355 PMC10323038

[2] J. Du , M. Carl , M. Bydder , A. Takahashi , C. B. Chung , and G. M. Bydder , “Qualitative and Quantitative Ultrashort Echo Time (UTE) Imaging of Cortical Bone,” Journal of Magnetic Resonance 207, no. 2 (2010): 304–311, 10.1016/j.jmr.2010.09.013. 20980179

[3] M. Weiger and K. P. Pruessmann , “MRI With Zero Echo Time,” in Encyclopedia of Magnetic Resonance, ed. R. K. Harris (John Wiley & Sons, Ltd, 2012), emrstm1292, 10.1002/9780470034590.emrstm1292.

[4] M. Weiger and K. P. Pruessmann , “Short‐T2 MRI: Principles and Recent Advances,” Progress in Nuclear Magnetic Resonance Spectroscopy 114‐115 (2019): 237–270, 10.1016/j.pnmrs.2019.07.001. 31779882

[5] E. Deininger‐Czermak , D. Gascho , S. Franckenberg , et al., “Added Value of Ultra‐Short Echo Time and Fast Field Echo Using Restricted Echo‐Spacing MR Imaging in the Assessment of the Osseous Cervical Spine,” La Radiologia Medica 128, no. 2 (2023): 234–241, 10.1007/s11547-023-01589-7. 36637741 PMC9938813

[6] Ü. Aydıngöz , A. E. Yıldız , and F. B. Ergen , “Zero Echo Time Musculoskeletal MRI: Technique, Optimization, Applications, and Pitfalls,” Radiographics 42, no. 5 (2022): 1398–1414, 10.1148/rg.220029. 35904982

[7] M. C. Florkow , K. Willemsen , V. V. Mascarenhas , E. H. G. Oei , M. Van Stralen , and P. R. Seevinck , “Magnetic Resonance Imaging Versus Computed Tomography for Three‐Dimensional Bone Imaging of Musculoskeletal Pathologies: A Review,” Journal of Magnetic Resonance Imaging 56, no. 1 (2022): 11–34, 10.1002/jmri.28067. 35044717 PMC9305220

[8] M. H. G. Dremmen , M. W. Wagner , T. Bosemani , et al., “Does the Addition of a “Black Bone” Sequence to a Fast Multisequence Trauma MR Protocol Allow MRI to Replace CT After Traumatic Brain Injury in Children?,” AJNR. American Journal of Neuroradiology 38, no. 11 (2017): 2187–2192, 10.3174/ajnr.A5405. 28970241 PMC7963587

[9] F. Altahawi and N. Subhas , “3D MRI in Musculoskeletal Imaging: Current and Future Applications,” Current Radiology Reports 6, no. 8 (2018): 27, 10.1007/s40134-018-0287-3.

[10] A. F. Lombardi , Y. J. Ma , H. Jang , et al., “Synthetic CT in Musculoskeletal Disorders: A Systematic Review,” Investigative Radiology 58, no. 1 (2023): 43–59, 10.1097/RLI.0000000000000916. 36070535 PMC9742139

[11] B. Johnson , H. Alizai , and M. Dempsey , “Fast Field Echo Resembling a CT Using Restricted Echo‐Spacing (FRACTURE): A Novel MRI Technique With Superior Bone Contrast,” Skeletal Radiology 50, no. 8 (2021): 1705–1713, 10.1007/s00256-020-03659-7. 33175183

[12] D. Lee , E. Kim , H. Woo , C. Y. Jeon , J. Yoon , and J. Choi , “Fast Field Echo Resembling CT Using Restricted Echo‐Spacing (FRACTURE) MR Sequence Can Provide Craniocervical Region Images Comparable to a CT in Dogs,” Frontiers in Bioengineering and Biotechnology 12 (2024): 1297675, 10.3389/fbioe.2024.1297675. 38476967 PMC10927716

[13] P. Malloy , J. Gasienica , R. Dawe , et al., “1.5 T Magnetic Resonance Imaging Generates Accurate 3D Proximal Femoral Models: Surgical Planning Implications for Femoroacetabular Impingement,” Journal of Orthopaedic Research 38, no. 9 (2020): 2050–2056, 10.1002/jor.24596. 31976569

[14] M. Samim , N. Eftekhary , J. M. Vigdorchik , et al., “3D‐MRI Versus 3D‐CT in the Evaluation of Osseous Anatomy in Femoroacetabular Impingement Using Dixon 3D FLASH Sequence,” Skeletal Radiology 48, no. 3 (2019): 429–436, 10.1007/s00256-018-3049-7. 30182297

[15] T. Finkenstaedt , P. Siriwanarangsun , S. Achar , et al., “Ultrashort Time‐To‐Echo Magnetic Resonance Imaging at 3 T for the Detection of Spondylolysis in Cadaveric Spines: Comparison With CT,” Investigative Radiology 54, no. 1 (2019): 32–38, 10.1097/RLI.0000000000000506. 30157099 PMC6269191

[16] E. C. Ang , A. F. Robertson , F. A. Malara , et al., “Diagnostic Accuracy of 3‐T Magnetic Resonance Imaging With 3D T1 VIBE Versus Computer Tomography in Pars Stress Fracture of the Lumbar Spine,” Skeletal Radiology 45, no. 11 (2016): 1533–1540, 10.1007/s00256-016-2475-7. 27614965

[17] B. J. Schwaiger , C. Schneider , S. Kronthaler , et al., “CT‐Like Images Based on T1 Spoiled Gradient‐Echo and Ultra‐Short Echo Time MRI Sequences for the Assessment of Vertebral Fractures and Degenerative Bone Changes of the Spine,” European Radiology 31, no. 7 (2021): 4680–4689, 10.1007/s00330-020-07597-9. 33443599 PMC8213670

[18] A. S. Gersing , D. Pfeiffer , F. K. Kopp , et al., “Evaluation of MR‐Derived CT‐Like Images and Simulated Radiographs Compared to Conventional Radiography in Patients With Benign and Malignant Bone Tumors,” European Radiology 29, no. 1 (2019): 13–21, 10.1007/s00330-018-5450-y. 29948069

[19] P. M. Jungmann , T. Lange , M. Wenning , F. A. Baumann , F. Bamberg , and M. Jung , “Ankle Sprains in Athletes: Current Epidemiological, Clinical and Imaging Trends,” Open access Journal of Sports Medicine 14 (2023): 29–46, 10.2147/OAJSM.S397634. 37252646 PMC10216848

[20] L. R. Chong , K. Lee , and F. Y. Sim , “3D MRI With CT‐Like Bone Contrast – An Overview of Current Approaches and Practical Clinical Implementation,” European Journal of Radiology 143 (2021): 109915, 10.1016/j.ejrad.2021.109915. 34461599

[21] E. Deininger‐Czermak , A. Euler , S. Franckenberg , et al., “Evaluation of Ultrashort Echo‐Time (UTE) and Fast‐Field‐Echo (FRACTURE) Sequences for Skull Bone Visualization and Fracture Detection – A Postmortem Study,” Journal of Neuroradiology 49, no. 3 (2022): 237–243, 10.1016/j.neurad.2021.11.001. 34758365

[22] S. Ryu , S. Park , E. Kim , et al., “Fast Field Echo Resembling a CT Using Restricted Echo‐Spacing (FRACTURE) Sequence for Shoulder Joint in Normal Dogs,” Frontiers in Veterinary Science 11 (2024): 1298133, 10.3389/fvets.2024.1298133. 38352037 PMC10861672

[23] E. Koh , E. R. Walton , and P. Watson , “VIBE MRI: An Alternative to CT in the Imaging of Sports‐Related Osseous Pathology?,” British Journal of Radiology 91, no. 1088 (2018): 20170815, 10.1259/bjr.20170815. 29474097 PMC6209485

[24] S. Gyftopoulos , A. Yemin , T. Mulholland , et al., “3DMR Osseous Reconstructions of the Shoulder Using a Gradient‐Echo Based Two‐Point Dixon Reconstruction: A Feasibility Study,” Skeletal Radiology 42, no. 3 (2013): 347–352, 10.1007/s00256-012-1489-z. 22829026

[25] S. Gyftopoulos , L. S. Beltran , A. Yemin , et al., “Use of 3D MR Reconstructions in the Evaluation of Glenoid Bone Loss: A Clinical Study,” Skeletal Radiology 43, no. 2 (2014): 213–218, 10.1007/s00256-013-1774-5. 24318071

[26] D. A. Lansdown , G. L. Cvetanovich , N. N. Verma , et al., “Automated 3‐Dimensional Magnetic Resonance Imaging Allows for Accurate Evaluation of Glenoid Bone Loss Compared With 3‐Dimensional Computed Tomography,” Arthroscopy: The Journal of Arthroscopic & Related Surgery 35, no. 3 (2019): 734–740, 10.1016/j.arthro.2018.10.119. 30733040

[27] C. A. Chen , R. Kijowski , L. M. Shapiro , et al., “Cartilage Morphology at 3.0T: Assessment of Three‐Dimensional Magnetic Resonance Imaging Techniques,” Journal of Magnetic Resonance Imaging 32, no. 1 (2010): 173–183, 10.1002/jmri.22213. 20578024 PMC3065186

[28] R. Kijowski and G. E. Gold , “Routine 3D Magnetic Resonance Imaging of Joints,” Journal of Magnetic Resonance Imaging 33, no. 4 (2011): 758–771, 10.1002/jmri.22342. 21448939 PMC3069719

[29] A. Guermazi , F. W. Roemer , H. Alizai , et al., “State of the Art: MR Imaging After Knee Cartilage Repair Surgery,” Radiology 277, no. 1 (2015): 23–43, 10.1148/radiol.2015141146. 26402492

[30] F. F. Altahawi , K. J. Blount , N. P. Morley , E. Raithel , and I. M. Omar , “Comparing an Accelerated 3D Fast Spin‐Echo Sequence (CS‐SPACE) for Knee 3‐T Magnetic Resonance Imaging With Traditional 3D Fast Spin‐Echo (SPACE) and Routine 2D Sequences,” Skeletal Radiology 46, no. 1 (2017): 7–15, 10.1007/s00256-016-2490-8. 27744578

[31] O. M. Abdulaal , L. Rainford , P. MacMahon , et al., “3T MRI of the Knee With Optimised Isotropic 3D Sequences: Accurate Delineation of Intra‐Articular Pathology Without Prolonged Acquisition Times,” European Radiology 27, no. 11 (2017): 4563–4570, 10.1007/s00330-017-4816-x. 28432504

[32] G. C. Feuerriegel , S. Kronthaler , K. Weiss , et al., “Assessment of Glenoid Bone Loss and Other Osseous Shoulder Pathologies Comparing MR‐Based CT‐Like Images With Conventional CT,” European Radiology 33, no. 12 (2023): 8617–8626, 10.1007/s00330-023-09939-9. 37453986 PMC10667374

[33] J. E. Vandevenne , F. Vanhoenacker , J. M. Mahachie John , G. Gelin , and P. M. Parizel , “Fast MR Arthrography Using VIBE Sequences to Evaluate the Rotator Cuff,” Skeletal Radiology 38, no. 7 (2009): 669–674, 10.1007/s00256-009-0677-y. 19294378

[34] C. Tian , Y. Shang , and Z. Zheng , “Glenoid Bone Lesions: Comparison Between 3D VIBE Images in MR Arthrography and Nonarthrographic MSCT,” Journal of Magnetic Resonance Imaging 36, no. 1 (2012): 231–236, 10.1002/jmri.23622. 22359387

[35] L. Stillwater , J. Koenig , B. Maycher , and M. Davidson , “3D‐MR vs. 3D‐CT of the Shoulder in Patients With Glenohumeral Instability,” Skeletal Radiology 46, no. 3 (2017): 325–331, 10.1007/s00256-016-2559-4. 28028575

[36] N. Wang , Z. Jin , F. Liu , et al., “Bone Injury Imaging in Knee and Ankle Joints Using Fast‐Field‐Echo Resembling a CT Using Restricted Echo‐Spacing MRI: A Feasibility Study,” Frontiers in Endocrinology 15 (2024): 15, 10.3389/fendo.2024.1421876. PMC11273369 39072275

[37] P. Rai and A. K. Janu , “Synthetic CT Demonstrates Multiple Enchondromas in Ollier Syndrome,” Radiology 314, no. 1 (2025): e241650, 10.1148/radiol.241650. 39772794

[38] S. Haller , E. M. Haacke , M. M. Thurnher , and F. Barkhof , “Susceptibility‐Weighted Imaging: Technical Essentials and Clinical Neurologic Applications,” Radiology 299, no. 1 (2021): 3–26, 10.1148/radiol.2021203071. 33620291

[39] E. M. Haacke , Y. Xu , Y. C. N. Cheng , and J. R. Reichenbach , “Susceptibility Weighted Imaging (SWI),” Magnetic Resonance in Medicine 52, no. 3 (2004): 612–618, 10.1002/mrm.20198. 15334582

[40] S. M. Böker , L. C. Adams , U. L. Fahlenkamp , G. Diederichs , B. Hamm , and M. R. Makowski , “Value of Susceptibility‐Weighted Imaging for the Assessment of Angle Measurements Reflecting Hip Morphology,” Scientific Reports 10 (2020): 20899, 10.1038/s41598-020-77671-1. 33262372 PMC7708417

[41] S. T. Ulas , K. Ziegeler , S. T. Richter , et al., “CT‐Like Images in MRI Improve Specificity of Erosion Detection in Patients With Hand Arthritis: A Diagnostic Accuracy Study With CT as Standard of Reference,” RMD Open 8, no. 1 (2022): e002089, 10.1136/rmdopen-2021-002089. 35177555 PMC8860086

[42] D. Nörenberg , M. Armbruster , Y. N. Bender , et al., “Diagnostic Performance of Susceptibility‐Weighted Magnetic Resonance Imaging for the Assessment of Sub‐Coracoacromial Spurs Causing Subacromial Impingement Syndrome,” European Radiology 27, no. 3 (2017): 1286–1294, 10.1007/s00330-016-4441-0. 27287483

[43] D. Deppe , K. G. Hermann , F. Proft , et al., “CT‐Like Images of the Sacroiliac Joint Generated From MRI Using Susceptibility‐Weighted Imaging (SWI) in Patients With Axial Spondyloarthritis,” RMD Open 7, no. 2 (2021): e001656, 10.1136/rmdopen-2021-001656. 34049998 PMC8166621

[44] S. M. Böker , L. C. Adams , Y. Y. Bender , et al., “Evaluation of Vertebral Body Fractures Using Susceptibility‐Weighted Magnetic Resonance Imaging,” European Radiology 28, no. 5 (2018): 2228–2235, 10.1007/s00330-017-5195-z. 29260364

[45] G. Engel , Y. Y. Bender , L. C. Adams , et al., “Evaluation of Osseous Cervical Foraminal Stenosis in Spinal Radiculopathy Using Susceptibility‐Weighted Magnetic Resonance Imaging,” European Radiology 29, no. 4 (2019): 1855–1862, 10.1007/s00330-018-5769-4. 30324384

[46] S. M. Böker , Y. Y. Bender , L. C. Adams , et al., “Evaluation of Sclerosis in Modic Changes of the Spine Using Susceptibility‐Weighted Magnetic Resonance Imaging,” European Journal of Radiology 88 (2017): 148–154, 10.1016/j.ejrad.2016.12.024. 28189200

[47] Y. Y. N. Bender , G. Diederichs , T. C. Walter , et al., “Differentiation of Osteophytes and Disc Herniations in Spinal Radiculopathy Using Susceptibility‐Weighted Magnetic Resonance Imaging,” Investigative Radiology 52, no. 2 (2017): 75–80, 10.1097/RLI.0000000000000314. 27548342

[48] S. M. Böker , L. C. Adams , Y. Y. Bender , et al., “Differentiation of Predominantly Osteoblastic and Osteolytic Spine Metastases by Using Susceptibility‐Weighted MRI,” Radiology 290, no. 1 (2019): 146–154, 10.1148/radiol.2018172727. 30375926

[49] M. Weiger , D. O. Brunner , B. E. Dietrich , C. F. Müller , and K. P. Pruessmann , “ZTE imaging in humans,” Magnetic Resonance in Medicine 70, no. 2 (2013): 328–332, 10.1002/mrm.24816. 23776142

[50] M. D. Robson , P. D. Gatehouse , M. Bydder , and G. M. Bydder , “Magnetic Resonance: An Introduction to Ultrashort TE (UTE) Imaging,” Journal of Computer Assisted Tomography 27, no. 6 (2003): 825–846, 10.1097/00004728-200311000-00001. 14600447

[51] J. Rahmer , P. Börnert , J. Groen , and C. Bos , “Three‐Dimensional Radial Ultrashort Echo‐Time Imaging With T2 Adapted Sampling,” Magnetic Resonance in Medicine 55, no. 5 (2006): 1075–1082, 10.1002/mrm.20868. 16538604

[52] M. Carl , G. M. Bydder , and J. Du , “UTE Imaging With Simultaneous Water and Fat Signal Suppression Using a Time‐Efficient Multispoke Inversion Recovery Pulse Sequence,” Magnetic Resonance in Medicine 76, no. 2 (2016): 577–582, 10.1002/mrm.25823. 26309221 PMC4769116

[53] A. M. Afsahi , Y. Ma , H. Jang , et al., “Ultrashort Echo Time Magnetic Resonance Imaging Techniques: Met and Unmet Needs in Musculoskeletal Imaging,” Journal of Magnetic Resonance Imaging 55, no. 6 (2022): 1597–1612, 10.1002/jmri.28032. 34962335 PMC9106865

[54] M. Weiger , K. P. Pruessmann , A. K. Bracher , et al., “High‐Resolution ZTE Imaging of Human Teeth,” NMR in Biomedicine 25, no. 10 (2012): 1144–1151, 10.1002/nbm.2783. 22290744

[55] D. Idiyatullin , C. Corum , J. Y. Park , and M. Garwood , “Fast and Quiet MRI Using a Swept Radiofrequency,” Journal of Magnetic Resonance (San Diego, Calif) 1997 181, no. 2 (2006): 342–349, 10.1016/j.jmr.2006.05.014. 16782371

[56] A. Techawiboonwong , H. K. Song , M. B. Leonard , and F. W. Wehrli , “Cortical Bone Water: In Vivo Quantification With Ultrashort Echo‐Time MR Imaging,” Radiology 248, no. 3 (2008): 824–833, 10.1148/radiol.2482071995. 18632530 PMC2798093

[57] M. Weiger , M. Stampanoni , and K. P. Pruessmann , “Direct Depiction of Bone Microstructure Using MRI With Zero Echo Time,” Bone 54, no. 1 (2013): 44–47, 10.1016/j.bone.2013.01.027. 23356986

[58] S. Jerban , Y. Ma , J. H. Wong , et al., “Ultrashort Echo Time Magnetic Resonance Imaging (UTE‐MRI) of Cortical Bone Correlates Well With Histomorphometric Assessment of Bone Microstructure,” Bone 123 (2019): 8–17, 10.1016/j.bone.2019.03.013. 30877070 PMC6504977

[59] S. Jerban , Y. Ma , A. Nazaran , et al., “Detecting Stress Injury (Fatigue Fracture) in Fibular Cortical Bone Using Quantitative Ultrashort Echo Time‐Magnetization Transfer (UTE‐MT): An Ex Vivo Study,” NMR in Biomedicine 31, no. 11 (2018): e3994, 10.1002/nbm.3994. 30059184 PMC6553877

[60] R. E. Breighner , Y. Endo , G. P. Konin , L. V. Gulotta , M. F. Koff , and H. G. Potter , “Technical Developments: Zero Echo Time Imaging of the Shoulder: Enhanced Osseous Detail by Using MR Imaging,” Radiology 286, no. 3 (2018): 960–966, 10.1148/radiol.2017170906. 29117482

[61] U. U. Bharadwaj , A. Coy , D. Motamedi , et al., “CT‐Like MRI: A Qualitative Assessment of ZTE Sequences for Knee Osseous Abnormalities,” Skeletal Radiology 51, no. 8 (2022): 1585–1594, 10.1007/s00256-021-03987-2. 35088162 PMC9198000

[62] R. E. Breighner , E. A. Bogner , S. C. Lee , M. F. Koff , and H. G. Potter , “Evaluation of Osseous Morphology of the Hip Using Zero Echo Time Magnetic Resonance Imaging,” American Journal of Sports Medicine 47, no. 14 (2019): 3460–3468, 10.1177/0363546519878170. 31633993

[63] M. E. Sahr , R. E. Breighner , A. J. Burge , et al., “Utility of Zero Echo Time MRI for the Diagnosis and Characterization of Ankle Fractures,” HSS Journal®: The Musculoskeletal Journal of Hospital for Special Surgery 20, no. 4 (2024): 502–507, 10.1177/15563316231187383. 39464656 PMC11512459

[64] A. Fujisaki , J. Tsukamoto , H. Narimatsu , et al., “Zero Echo Time Magnetic Resonance Imaging; Techniques and Clinical Utility in Musculoskeletal System,” Journal of Magnetic Resonance Imaging 59, no. 1 (2024): 32–42, 10.1002/jmri.28843. 37288953

[65] C. Lee , K. J. Jeon , S. S. Han , et al., “CT‐Like MRI Using the Zero‐TE Technique for Osseous Changes of the TMJ,” Dento Maxillo Facial Radiology 49, no. 3 (2020): 20190272, 10.1259/dmfr.20190272. 31670578 PMC7068078

[66] Y. E. Bayrak , T. Özer , Y. Anik , et al., “Assessment of Sacroiliitis Using Zero Echo Time Magnetic Resonance Imaging: A Comprehensive Evaluation,” Pediatric Radiology (2025), 10.1007/s00247-025-06201-w. PMC12065742 39998585

[67] V. Vuillemin , H. Guerini , F. Thévenin , et al., “Bone Tissue in Magnetic Resonance Imaging: Contribution of New Zero Echo Time Sequences,” Seminars in Musculoskeletal Radiology 27, no. 4 (2023): 411–420, 10.1055/s-0043-1770771. 37748464

[68] L. Wolharn , R. Guggenberger , K. Higashigaito , et al., “Detailed Bone Assessment of the Sacroiliac Joint in a Prospective Imaging Study: Comparison Between Computed Tomography, Zero Echo Time, and Black Bone Magnetic Resonance Imaging,” Skeletal Radiology 51, no. 12 (2022): 2307–2315, 10.1007/s00256-022-04097-3. 35773420 PMC9560917

[69] Z. Zhang , J. Wang , Y. Li , et al., “Bone Assessment of the Sacroiliac Joint in Ankylosing Spondylitis: Comparison Between Computed Tomography and Zero Echo Time MRI,” European Journal of Radiology 181 (2024): 111743, 10.1016/j.ejrad.2024.111743. 39341167

[70] Y. Li , Y. Xiong , B. Hou , et al., “Comparison of Zero Echo Time MRI With T1‐Weighted Fast Spin Echo for the Recognition of Sacroiliac Joint Structural Lesions Using CT as the Reference Standard,” European Radiology 32, no. 6 (2022): 3963–3973, 10.1007/s00330-021-08513-5. 35059805

[71] C. V. Tran , H. R. Yang , Z. Y. Ahmad , et al., “Utility of Zero‐Echo Time Sequence as an Adjunct for MR Evaluation of Degenerative Disease in the Cervical Spine,” Skeletal Radiology 53, no. 5 (2024): 899–908, 10.1007/s00256-023-04507-0. 37945769

[72] E. C. Argentieri , M. F. Koff , R. E. Breighner , Y. Endo , P. H. Shah , and D. B. Sneag , “Diagnostic Accuracy of Zero‐Echo Time MRI for the Evaluation of Cervical Neural Foraminal Stenosis,” Spine 43, no. 13 (2018): 928–933, 10.1097/BRS.0000000000002462. 29095415

[73] T. Caffard , E. Chiapparelli , A. Arzani , et al., “Diagnosis and Evaluation of Cervical Ossification of the Posterior Longitudinal Ligament on Zero‐Echo Time Magnetic Resonance Imaging: An Illustrative Case Series,” European Journal of Orthopaedic Surgery & Traumatology 35, no. 1 (2024): 9, 10.1007/s00590-024-04116-0. 39567399

[74] B. Hou , C. Liu , Y. Li , et al., “Evaluation of the Degenerative Lumbar Osseous Morphology Using Zero Echo Time Magnetic Resonance Imaging (ZTE‐MRI),” European Spine Journal 31, no. 3 (2022): 792–800, 10.1007/s00586-021-07099-2. 35015138

[75] F. E. Lecouvet , D. Zan , D. Lepot , et al., “MRI‐Based Zero Echo Time and Black Bone Pseudo‐CT Compared With Whole‐Body CT to Detect Osteolytic Lesions in Multiple Myeloma,” Radiology 313, no. 1 (2024): e231817, 10.1148/radiol.231817. 39377681

[76] Y. Xu , L. Shi , N. Li , J. Meng , Q. Wang , and H. Wang , “Value of Zero Echo Time MR Imaging and CT in Diagnosis of Bone Destructions of Bone Tumors and Tumor‐Like Lesions,” Chinese Journal of Academic Radiology 3, no. 2 (2020): 108–114, 10.1007/s42058-020-00035-1.

[77] H. S. Qassim and M. A. Kadhim , “Role of MRI Zero Echo Time in the Evaluation of Bone Lesions,” Iraqi Postgraduate Medical Journal 22, no. 3 (2023): 261–266, 10.52573/ipmj.2023.181131.

[78] J. Antunes , T. Young , D. Pittock , et al., “Assessing Multiple MRI Sequences in Deep Learning‐Based Synthetic CT Generation for MR‐Only Radiation Therapy of Head and Neck Cancers,” Radiotherapy and Oncology 205 (2025): 110782, 10.1016/j.radonc.2025.110782. 39929288

[79] A. P. Leynes , J. Yang , D. D. Shanbhag , et al., “Hybrid ZTE/Dixon MR‐Based Attenuation Correction for Quantitative Uptake Estimation of Pelvic Lesions in PET/MRI,” Medical Physics 44, no. 3 (2017): 902–913, 10.1002/mp.12122. 28112410 PMC5877454

[80] G. Delso , F. Wiesinger , L. I. Sacolick , et al., “Clinical Evaluation of Zero‐Echo‐Time MR Imaging for the Segmentation of the Skull,” Journal of Nuclear Medicine 56, no. 3 (2015): 417–422, 10.2967/jnumed.114.149997. 25678489

[81] P. A. G. Teixeira , H. Kessler , L. Morbée , et al., “Mineralized Tissue Visualization With MRI: Practical Insights and Recommendations for Optimized Clinical Applications,” Diagnostic and Interventional Imaging S2211–5684, no. 24 (2024): 00256‐0, 10.1016/j.diii.2024.11.001. 39667997

[82] F. Wiesinger and M. L. Ho , “Zero‐TE MRI: Principles and Applications in the Head and Neck,” British Journal of Radiology 95, no. 1136 (2022): 20220059, 10.1259/bjr.20220059. 35616709 PMC10162052

[83] M. Buschmann , H. Herrmann , M. Gober , et al., “Development of an MR‐Only Radiotherapy Treatment Planning Workflow Using a Commercial Synthetic CT Generator for Brain and Head & Neck Tumor Patients,” Zeitschrift für Medizinische Physik (2025): S0939388925000285, 10.1016/j.zemedi.2025.01.003.39956751

[84] A. K. Parchur , M. Zarenia , C. Gage , E. S. Paulson , and E. Ahunbay , “Automated Hallucination Detection for Synthetic CT Images Used in MR‐Only Radiotherapy Workflows,” Physics in Medicine and Biology 70, no. 5 (2025): 05NT01, 10.1088/1361-6560/adb5eb. 39946843

[85] T. T. Y. Yip , Z. Li , and T. Li , “Clinical Validation of MR‐Generated Synthetic CT by MRCAT for Brain Tumor Radiotherapy,” Journal of Applied Clinical Medical Physics 26, no. 1 (2025): e14494, 10.1002/acm2.14494. 39673148 PMC11713499

[86] M. Fusella , E. Alvarez Andres , F. Villegas , et al., “Results of 2023 Survey on the Use of Synthetic Computed Tomography for Magnetic Resonance Imaging‐Only Radiotherapy: Current Status and Future Steps,” Physics and Imaging in Radiation Oncology 32 (2024): 100652, 10.1016/j.phro.2024.100652. 39381612 PMC11460247

[87] C. Lee , Y. H. Yoon , J. Sung , et al., “Abdominal Synthetic CT Generation for MR‐Only Radiotherapy Using Structure‐Conserving Loss and Transformer‐Based Cycle‐GAN,” Frontiers in Oncology 14 (2025): 1478148, 10.3389/fonc.2024.1478148. 39830649 PMC11739088

[88] S. H. Park , D. M. Choi , I. H. Jung , et al., “Clinical Application of Deep Learning‐Based Synthetic CT From Real MRI to Improve Dose Planning Accuracy in Gamma Knife Radiosurgery: A Proof of Concept Study,” Biomedical Engineering Letters 12, no. 4 (2022): 359–367, 10.1007/s13534-022-00227-x. 36238366 PMC9550914

[89] L. Morbée , M. Chen , N. Herregods , P. Pullens , and L. B. O. Jans , “MRI‐Based Synthetic CT of the Lumbar Spine: Geometric Measurements for Surgery Planning in Comparison With CT,” European Journal of Radiology 144 (2021): 109999, 10.1016/j.ejrad.2021.109999. 34700094

[90] M. C. Florkow , K. Willemsen , F. Zijlstra , et al., “MRI‐Based Synthetic CT Shows Equivalence to Conventional CT for the Morphological Assessment of the Hip Joint,” Journal of Orthopaedic Research 40, no. 4 (2022): 954–964, 10.1002/jor.25127. 34191351 PMC9291600

[91] L. B. O. Jans , M. Chen , D. Elewaut , et al., “MRI‐Based Synthetic CT in the Detection of Structural Lesions in Patients With Suspected Sacroiliitis: Comparison With MRI,” Radiology 298, no. 2 (2021): 343–349, 10.1148/radiol.2020201537. 33350891

[92] J. Upadhyay , J. Iwasaka‐Neder , E. Golden , and S. Bixby , “Synthetic CT Assessment of Lesions in Children With Rare Musculoskeletal Diseases,” Pediatrics 152, no. 2 (2023): e2022061027, 10.1542/peds.2022-061027. 37416976 PMC10389770

[93] N. Kamona , B. C. Jones , H. Lee , et al., “Cranial Bone Imaging Using Ultrashort Echo‐Time Bone‐Selective MRI as an Alternative to Gradient‐Echo Based “Black‐Bone” Techniques,” Magnetic Resonance Materials in Physics, Biology and Medicine 37, no. 1 (2023): 83–92, 10.1007/s10334-023-01125-8. PMC10923077 37934295

[94] A. Lecler , L. Duron , E. Charlson , et al., “Comparison Between 7 Tesla and 3 Tesla MRI for Characterizing Orbital Lesions,” Diagnostic and Interventional Imaging 103, no. 9 (2022): 433–439, 10.1016/j.diii.2022.03.007. 35410799 PMC12498293

[95] F. Ensle , M. Kaniewska , M. Lohezic , and R. Guggenberger , “Enhanced Bone Assessment of the Shoulder Using Zero‐Echo Time MRI With Deep‐Learning Image Reconstruction,” Skeletal Radiology 53, no. 12 (2024): 2597–2606, 10.1007/s00256-024-04690-8. 38658419 PMC11493801

[96] L. Carretero‐Gómez , M. Fung , F. Wiesinger , et al., “Deep Learning‐Enhanced Zero Echo Time MRI for Glenohumeral Assessment in Shoulder Instability: A Comparative Study With CT,” Skeletal Radiology (2024), 10.1007/s00256-024-04830-0. PMC12000158 39572485

[97] K. Bae , K. N. Jeon , M. J. Hwang , et al., “Comparison of Lung Imaging Using Three‐Dimensional Ultrashort Echo Time and Zero Echo Time Sequences: Preliminary Study,” European Radiology 29, no. 5 (2019): 2253–2262, 10.1007/s00330-018-5889-x. 30547204

[98] K. Bae , J. Lee , Y. Jung , J. de Arcos , and K. N. Jeon , “Deep Learning Reconstruction for Zero Echo Time Lung Magnetic Resonance Imaging: Impact on Image Quality and Lesion Detection,” Clinical Radiology 79, no. 11 (2024): e1296–e1303, 10.1016/j.crad.2024.07.011. 39112100

[99] N. S. Higano , R. J. Fleck , D. R. Spielberg , et al., “Quantification of Neonatal Lung Parenchymal Density via Ultrashort Echo Time MRI With Comparison to CT,” Journal of Magnetic Resonance Imaging 46, no. 4 (2017): 992–1000, 10.1002/jmri.25643. 28160357 PMC6457694

[100] H. N. Ozcan , G. Ozer , H. S. Dogan , et al., “Zero‐Echo Time MRI: An Alternative Method for the Diagnosis of Urinary Stones in Children,” European Radiology 35, no. 1 (2025): 289–296, 10.1007/s00330-024-10950-x. 38992108 PMC11632057

